# Plaque Removal and Gingival Health after Use of a Novel Dental Gel: A Clinical Study

**DOI:** 10.4172/2161-1122.1000396

**Published:** 2016-10-18

**Authors:** Anuradha Nayudu, Tracie Lam, Jessica Ho, Ali Forghany, Thinh Vu, William Ngo, Janet Ajdaharian, Petra Wilder-Smith

**Affiliations:** Beckman Laser Institute, University of California, Irvine, CA

**Keywords:** Plaque, Gingival inflammation, Dentifrice, Oral hygiene, Dental gel

## Abstract

**Background:**

Goal of this *in vivo* prospective, randomized, controlled, double-blinded, cross over study was to compare the level of plaque control and gingivitis after use of a novel dental gel (test) vs. A Triclosan/copolymer dentifrice (control).

**Methods:**

After coronal polishing, 22 subjects with moderate gingivitis were randomly assigned to brush twice daily with test or control dentifrice for the first study Arm. Plaque, gingival and sulcus bleeding indices were recorded at baseline, week 2 and week 4. Professional coronal polishing was repeated, and then subjects brushed with the second dentifrice for 4 weeks. Clinical indices were again recorded at Baseline, week 2 and week 4. The effects of each dentifrice on clinical indices were compared using Student’s t-test.

**Results:**

Brushing with the test gel produced significantly greater levels of plaque reduction versus the Triclosan/copolymer control dentifrice at each time point. 45% less plaque was measured after 4 weeks of test agent use than after control agent use (p<0.000000005). A significant reduction in gingival inflammation from test vs control agent over w\4 weeks was also observed (p=0.000342).

**Conclusions:**

An activated edathamil dental gel formulation provides effective plaque control and reduced gingival inflammation compared to a Triclosan/Co-polymer dental gel. Practical Implications: A novel dental gel formulation that does not contain abrasives, detergents or antimicrobials may provide effective plaque control and support gingival health.

## Introduction

Despite heightened awareness, concerted public health measures and use of mechanical and chemical methods of plaque control, the prevalence of severe periodontal disease has not decreased in the US [1]. Based on data collected as part of CDC's 2009–2010 National Health and Nutrition Examination Survey (NHANES), 47.2% of adults aged 30 years and older in the US have some form of periodontal disease [2]. Prevalence and severity of periodontal disease increase with age and 70% of adults 65 years and older show signs and symptoms of this condition [2]. As Americans live longer, periodontal disease is affecting an increasing proportion of the population. In total, approximately 64.7 million adults suffer from periodontitis and 9% suffer from severe periodontitis [2].

Periodontitis is a chronic inflammatory condition that affects the integrity of the tooth-supporting tissues including the gingiva, periodontal ligament, and alveolar bone [3]. If left untreated, it leads to breakdown of the supporting structures, tooth loosening and eventual tooth loss. Typically, periodontitis is preceded by gingivitis, and poor oral hygiene is a common factor in patients with these conditions [4,5]. Risk factors for periodontal disease include cigarette smoking, diabetes mellitus, prediabetes, obesity, metabolic syndrome, osteoporosis and stress [6]. The prevalence of diabetes in US is 8%, obesity is at 36% and smoking is at 20%, and these populations account for a considerable proportion of the population that is at increased risk for developing periodontal disease [7].

Effective and consistent plaque control is a crucial step in preventing the progression of gingivitis to periodontitis in susceptible individuals, medically compromised patients, and also in patients with limited strength and manual dexterity, such as the elderly, the very young, or handicapped. Recent findings have also confirmed an extensive network of potential linkage between periodontal disease and systemic diseases such as cardiovascular disease, diabetes [8], adverse pregnancy outcomes [9], rheumatic arthritis [10], aspiration pneumonia and COPD [11]. Periodontal disease has also been implicated as a causational factor in colorectal cancer [12], oral squamous cell carcinoma [9], pancreatic cancer [13] and breast cancer [14].

The level of plaque control that is achieved through current state-of-the-art mechanical and chemical methods including tooth brushing, flossing, anti-bacterial or anti-plaque formulations leaves room for improvement [15]. Mechanical plaque removal depends heavily on patient compliance, and sometimes it is difficult to establish daily habits for arduous, repetitive and time-consuming habits like flossing and using interdental aids [15]. Thus there is interest in chemical anti-plaque formulations. Side-effects of such existing anti-plaque formulations can include taste alteration and staining of teeth [16]. Dentifrice formulations containing mild abrasives can increase the effectiveness of brushing, but, if they are used inexpertly, these can cause dental abrasion, sensitivity or gingival lesions [17]. The addition of pyrophosphate to dentifrice formulations has been shown to reduce crystal formation in supra gingival calculus but does not affect sub gingival calculus [17]. Clearly there exists a need for novel, more effective approaches to oral plaque control.

Calcium ions play a role in the binding and adhesion mechanisms involving the plaque material and plaque pellicle interface [18]. Previous research has demonstrated that formulations containing activated edathamil may achieve disruption of plaque without adversely affecting the enamel surface microstructure or recovery from acid challenge [19–21].

The goal of this in vivo prospective, randomized, controlled, double-blinded crossover study was to compare the level of plaque control achieved using an activated Edathamil dental gel (test) vs. A Triclosan/copolymer dentifrice (control). The activated edathamil formulation combines FDA GRAS (Generally Regarded as Safe) and natural ingredients in a soft dental gel The study was designed to test the hypothesis that the novel dental gel will be at least as effective as an existing OTC dentifrice in removing plaque and supporting gingival health.

## Materials and Methods

This project was performed in full compliance with University of California at Irvine IRB-approved protocol #2002–2805. Written informed consent was obtained from all participants prior to study begin.

### Subjects

Twenty two subjects in good general health ranging in age from 20–34 years old (mean age of 24 years) with mild to moderate gingival inflammation (Löe and Silness Gingival Index ≥2) [22] and all pocket depths ≤5 mm completed this prospective, randomized, controlled, double-blinded study. All subjects had received a professional dental cleaning 4–8 weeks prior to enrollment in this study. Seven subjects were female and 15 were male; 2 were Caucasian, 4 were Asian, 14 were Hispanic, 1 was South Asian and 1 was African American. Subjects were screened to exclude persons with any known history of allergy to personal care/consumer products or their ingredients, and to any ingredients in the test, control and washout products. Other exclusion criteria included any medical condition which requires premedication prior to dental procedures, any diseases of the soft or hard oral tissues, history of any systemic disease that could result in being immune compromised or delayed wound healing, and use of antibiotics within the last 3 months. Subjects with a known history of hepatitis, HIV, ulcer forming diseases, abscesses, granulomas, or severe gingivitis or periodontitis were also excluded. All participants were non-smokers and neither pregnant nor lactating.

### Clinical protocol

Subjects were randomly assigned to brush twice daily for 4 weeks with either the OTC test dental gel (LivionexR Dental Gel, Los Gatos, CA), or the OTC control gel (Colgate TotalR, Colgate-Palmolive, Piscataway N.J.). After four weeks, the treatment was reversed for another four weeks. Subjects that had brushed with the Test Gel in Arm 1 of the study now used the control gel and vice versa. A standard Oral B ProFlexR toothbrush was provided to each volunteer and subjects were trained in standard sulcular brushing technique. Use of any other oral hygiene measures was not permitted, included mouthwashes and chewing gum. At each visit this information was repeated to the subjects and a written information sheet was also sent home with them after each visit. Subjects brushed their teeth twice a day, once in the morning, and once before going to bed, including on the day of each visit and the subjects refrained from eating from the time of brushing until after their assessment visit. Plaque levels (Turesky Modification of Quigley-Hein Index [23] (P.I.), gingival inflammation (Löe and Silness Gingival Index [22] (G.I.), and sulcus bleeding (mSBI) [24] were recorded.

A standardized pressure sensitive probe (Florida Probe) with 20 g probing force used by 1 pre-standardized clinician was used to ensure standardized probing in each subject at each time point. Volunteers were evaluated at Baseline, 2 weeks, 4 weeks, 6 weeks and 8 weeks by the same blinded, pre-calibrated investigator. All supra-gingival plaque was removed by coronal polishing directly before baseline measurements, and after measurements at the 4 week visit at the end of Arm 1 of the study.

**Figure 1:**
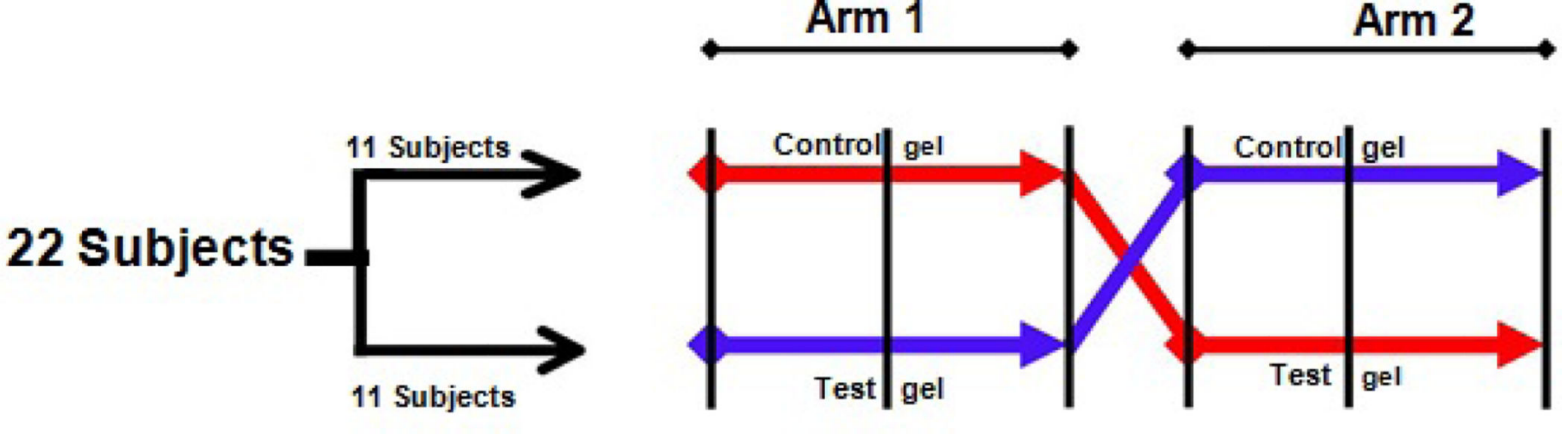
Flow chart of study design.

This removal of supra-gingival plaque substituted for a washout period. All investigators and subjects were blinded to the dental gel identity by the use of identical toothpaste tubes labeled only with a coded number. Only the study manager (who was not associated with the clinical evaluation) had access to the key for the sample codes. Subjects were monitored and questioned regarding any adverse effects at each visit and also provided with a direct telephone number to contact in case of any adverse effects (Figure 1).

### Measured indices

#### Quigley-Hein Plaque Index

Plaque was scored according to the Turesky modification of the Quigley-Hein Plaque Index. A score of 0–5 is assigned to each facial and lingual non-restored surface of all the teeth according to the following criteria:

0: No plaque.
1: Separate flecks of plaque at the cervical margin.
2: A thin, continuous band of plaque (up to 1 mm) at the cervical margin.
3: A band of plaque wider than 1 mm, but covering less than 1/3 of the side of the crown of the tooth.
4: Plaque covering at least 1/3, but less than 2/3 of the side of the crown of the tooth.
5: Plaque covering 2/3 or more of the side of the crown of the tooth.

#### Loe-silness gingival index

Each tooth was divided into two surfaces, facial and lingual. Those teeth with cervical restorations or prosthetic crowns were excluded from the scoring procedure. The gingiva adjacent to each tooth surface was scored as follows:

0: Absence of inflammation.
1: Mild inflammation: Slight change in color and little change in texture.
2: Moderate inflammation: Moderate glazing, redness, edema, and hypertrophy.
3: Severe inflammation: Marked redness and hypertrophy. Tendency for spontaneous bleeding.

#### Modified sulcus bleeding index

A score of 0–3 was assigned to each facial and lingual non-restored surface of all the teeth according to the following criteria:

0: No bleeding when periodontal probe is passed along the gingival margin.
1: Isolated bleeding spots visible.
2: Blood forms a confluent red line on the gingival margin.
3: Heavy or profuse bleeding.

## Results

**Figure 2:**
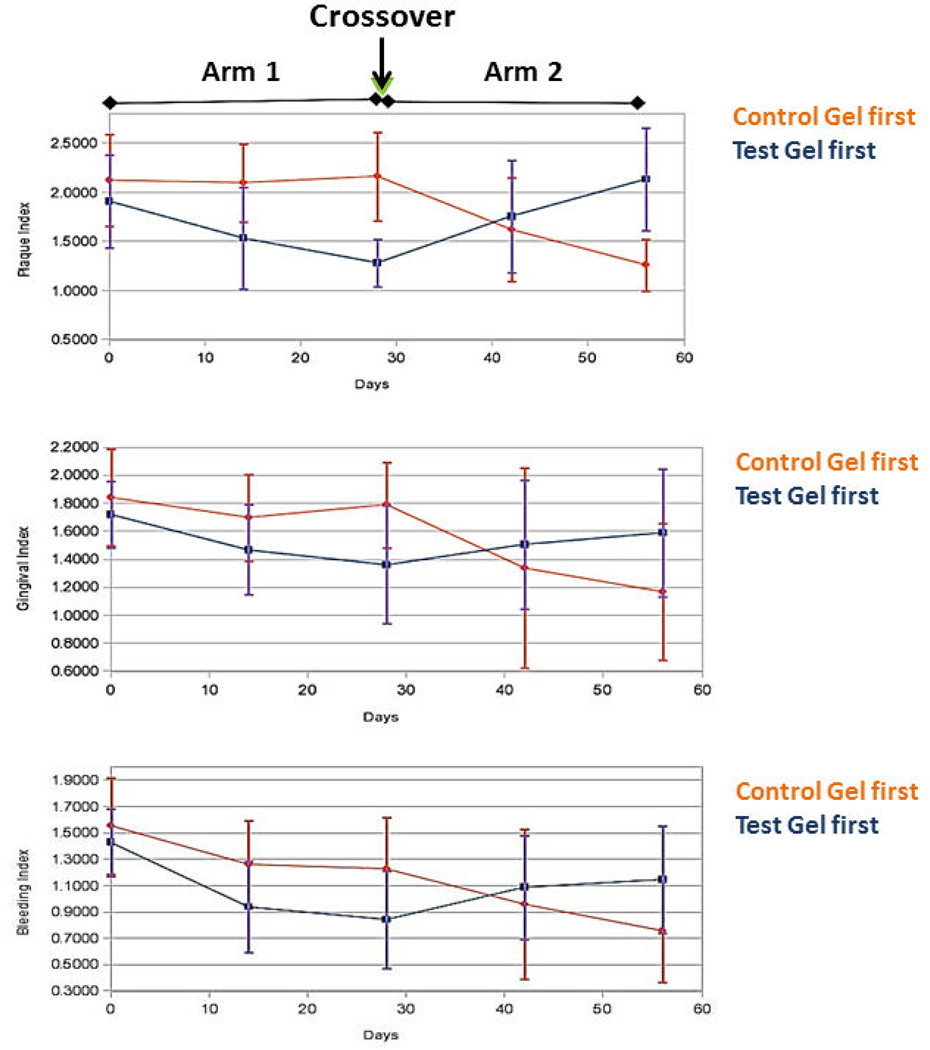
Mean clinical indices (S.D.) over the duration of the 8-week study. Arm 1 extended from day 0-day 28 (0–4 weeks) and Arm 2 extended from day 29–56 (5–8 weeks).

Clinical indices at baseline, 2 weeks, 4 weeks, 6 weeks and 8 weeks are recorded in Figure 2. At study outset, clinical indices were comparable in the 2 groups.

At baseline, P.I. averaged 1.99; mean G.I. measured 1.79, and mSBI averaged 1.48. Changes from the baseline value for each index were computed for each subject at the end of each Arm of the study (At 4 weeks and at 8 weeks.) and formed the basis of the comparison between the two treatments. These differences from baseline were analyzed according to the methodology suggested by Wellek and Blettner [25] for a two way crossover study. No significant carryover effect from the first Arm to the second was identified for any of the indices (p>0.328) (Table 1). For all of the clinical indices measured, test gel use achieved a greater improvement than brushing with the control gel (Table 2) (p<0.001). This effect was greatest with regard to plaque removal.

## Discussion

Goal of this *in vivo* study was to compare the level of plaque control and clinical gingivitis after the use of an Edathamil dental gel (test) vs. A Triclosan/copolymer dentifrice (control) dentifrice in twenty-two subjects with mild to moderate gingivitis. A professional prophylaxis 4–7 weeks prior to enrollment in the study ensured the absence of anything more than minimal calculus, as well as time for post-procedure gingival healing. Additional professional supra-gingival plaque removal and coronal polishing were instituted directly before baseline and after completion of Arm 1, directly before crossover to Arm 2, to eliminate any direct carry-over effect of each dentifrice.

The oral hygiene of the subjects as measured by the plaque index improved during the course of this study. Previous research has shown that this effect is not uncommon in studies that relate to oral hygiene, fueled in part by the prospects of frequent oral exams combined with motivation to perform well and provide useful information [26]. Additional variables that potentially may have contributed to variations in the amount of effort that subjects put into oral hygiene procedures over the course of the study could include a learning effect, boredom with the procedures, or increasing levels of focus on their personal oral hygiene [26,27]. A crossover study protocol is a valid design for study plaque removal efficacy where there are minimal carry over effects [26,27]. Furthermore, in order to minimize the effects of these variables in this study, toothpaste usage sequence was randomized so that one half of the subjects used the test gel in Arm 1, and the remaining 11 subjects used the control gel in Arm 1 [28]. Overall, during the 8-week study period, oral health improved in both groups.

When the control gel was used first, gingival inflammation and bleeding improved. However, this was accomplished without a corresponding reduction in the Plaque Index (Table 1). This observation is not unexpected, as triclosan is a bacteriocide that is effective against the bacteria that cause inflammation and bleeding [29], but not necessarily effective in combatting the oral biofilm itself [30]. Previous studies have reported mixed findings, identifying varying levels of antiplaque activity by the control gel [31,32]. One study over 6 months reported results similar to those determined in this study: A small reduction in supragingival plaque was associated with a significantly greater reduction in gingivitis (20%) and gingivitis severity (28%) [33]. After subjects crossed over to using the test gel in Arm 2 of the study, a reduction in the plaque index was observed in addition to a decline in gingival bleeding and inflammation. These results are supported by data from previous imaging and clinical studies which indicate that the test gel very effectively reduces plaque levels in the oral cavity, as well as inflammation and bleeding [34].

When the test gel was used first, all three clinical indices declined, supporting the findings of previous studies [35–37]. However after the crossover to the control gel, all three indices increased, with a greater relative rise in the plaque index than in the gingival and sulcus bleeding indices. While the increase in plaque might have been expected, based on information that the control dentifrice is most likely better at controlling bacteria than plaque biofilm [29,30], the increase in inflammation and bleeding was not. This finding might indicate that the bactericidal effects of the control formulation may not suffice to control the causative mechanisms of gingival inflammation when residual oral biofilm is present-even when the bacteria embedded in the plaque matrix are non-vital. Triclosan has an antimicrobial activity and promotes plaque control by disrupting the bacterial cell wall at a sub lethal concentration [4], however it has no known effect on the inorganic content of the plaque.

The lower plaque indices observed after use of the test gel in either Arm of the study support the initial hypothesis that the novel dental gel formulation may provide improved plaque control. The results were consistent with prior published studies that have used the test gel [34,38–40]. While some previous studies have demonstrated effective plaque inhibition by edathamil [41], other publications describe only a small anti-plaque effect, attributed to the limited ability of conventional edathamil formulations to penetrate biofilm [42]. To overcome this hurdle, a carrier and permeability enhancer to promote biofilm penetration and enhance anti-plaque efficacy are contained in the test dental gel used in this study [43].

Identifying novel means of plaque control is important because existing approaches have implications that extend far beyond reducing the prevalence of gingivitis and its progression to periodontitis. Recent studies have found an increased risk of endocrine disruption with the use of Triclosan and an association with thyroid disorders, allergies and antimicrobial resistance [44]. Also, dentifrice-containing triclosan relies on mechanical brushing action to disrupt the biofilm [45]. Although this modality can be very effective in removing plaque, some studies have found that it has a small effect in reducing gingivitis, particularly in high-risk individuals [45]. Also it heavily depends upon patient compliance and consistent, repeated effort, with limited effectiveness in areas of the mouth that are difficult to access, or in patients with painful oral conditions like burning mouth syndrome, oral lichen plans, or in patients undergoing orthodontic treatments [46].

In order to support the removal of inorganic plaque content through a mechanical scrubbing effect, many dentifrices contain abrasives. Long-term of the tooth surface to such agents, especially in combination with a zealous scrubbing motion often causes abrasions and tooth sensitivity, especially in patients with exposed root surfaces and in elderly patients with gingival recession [47]. Yet the prognosis and long-term success of periodontal surgery critically depend on effective plaque biofilm control [48]. Paradoxically, plaque biofilm control procedures that are crucial to preventing the recurrence or progression of periodontitis may themselves cause damage to the dental hard tissues.

Chemical plaque control presents an alternative option that potentially overcomes some of the challenges and limitations inherent to mechanical oral hygiene techniques. Chlorhexidine is the most commonly used example of this approach, providing excellent short-term plaque control. However, its limitations include tooth staining and taste alterations after longer usage [49]. Thus, there exists a critical need for novel approaches that can overcome these limitations and provide effective plaque control all persons, and especially in high-risk individuals, medically compromised patients and in periodontal patients. The novel plaque-control formulation used in this study may provide a means of overcoming the shortcomings of existing mechanical and chemical methods of plaque control.

## Conclusion

In this clinical study, a test dental gel demonstrated significantly more effective plaque control and reduced gingival inflammation compared to the control gel in an 8-week-long double-blinded two-Arm, two-treatment crossover study. Further studies are required to identify its effectiveness over longer periods of time and in patients with limited manual dexterity or with medical challenges.

## Figures and Tables

**Table 1:** Effect of gel use sequence on clinical indices. There was no significant carryover effect for any clinical index measurement parameter.

	Plaque Index		Bleeding Index		Gingival Index	
	T → C	C → T	T → C	C → T	T → C	C → T
**Mean**	−0.4045	−0.821	−0.8682	−1.126	−0.4912	−0.7261
**Std Dev**	1.0136	0.8589	0.703	0.6919	0.8077	0.5613
**N**	13	8	13	8	13	8
**T Statistic**	1.00373		0.08511		0.7531	
**df**	20		20		20	
**p value**	0.3275		0.4048		0.4602	
**Significanc** **e at p=0.05**	Not Significant		Not Significant		Not Significant	

**Table 2:** Effects of each gel on clinical indices. The test gel performed significantly better than the control gel for all parameters, considerably outperforming the control gel for plaque removal.

	Plaque Index		Bleeding Index		Gingival Index	
	T → C	C → T	T → C	C → T	T → C	C → T
**Mean index change**	−0.8496	0.9017	−0.3033	0.4692	−0.2293	0.6228
**Std Dev**	0.3885	0.315	0.512	0.4248	0.4946	0.3913
**N**	13	8	13	8	13	8
**T Statistic**	−11.192		−3.7186		−4.3085	
**df**	20		20		20	
**p value**	<0.000000005		0.00136		0.000342	
**Significance at p=0.05**	Significant		Significant		Significant	
